# Special Issue “Novel Therapeutic Targets in Cancers: 3rd Edition”

**DOI:** 10.3390/ijms27125288

**Published:** 2026-06-11

**Authors:** Elena Levantini, Giorgia Maroni

**Affiliations:** 1 CNR-Consiglio Nazionale delle Ricerche, Istituto di Tecnologie Biomediche (ITB-CNR), Area della Ricerca di Pisa, 56124 Pisa, PI, Italy; giorgia.maroni@itb.cnr.it; 2 Fondazione Pisana per la Scienza ONLUS (FPS), 56017 San Giuliano Terme, PI, Italy

Cancer remains one of the most complex and adaptive diseases in clinical medicine, characterized by remarkable genetic and functional heterogeneity across patients, tumor types, and even within individual lesions. This diversity underlies tumor progression and metastatic dissemination and, critically, fuels the emergence of therapy resistance, a persistent obstacle that continues to limit the long-term efficacy of current therapeutic strategies.

Yet, describing cancer merely as a genetic disease remains conceptually insufficient. Beyond a simple accumulation of mutations, cancer should be viewed as a dynamic and adaptive system, continuously shaped by the interplay between genomic instability, regulatory networks, and environmental pressures. This distinction is not merely semantic; it fundamentally reframes how therapeutic vulnerabilities are identified and exploited.

In this context, we conceived this Special Issue as an opportunity to capture the evolving logic of cancer biology in an era increasingly driven by systems-level investigation. While precision oncology has transformed our ability to dissect tumor genomes, growing evidence indicates that tumor vulnerability rarely resides in isolated genes but instead emerges from transient states within highly interconnected biological systems.

In this view, cancer is not simply resistant: it is *adaptively resilient*. Tumor cells continuously rewire their internal circuitry in response to therapeutic pressure, stabilizing phenotypic states that sustain growth, dissemination, and relapse. As a result, effective therapeutic strategies must move beyond single-target inhibition toward disruption of the dynamic systems that enable adaptation.

As conceptually illustrated in Figure 1, tumor behavior emerges from the dynamic interplay of interconnected regulatory layers operating under continuous selective pressure. Rather than a static collection of genetic alterations, cancer is better understood as a dynamic and adaptive system. Within this adaptive landscape, continuous network rewiring drives transitions between phenotypic states, ultimately giving rise to context-dependent vulnerabilities.

The schematic illustrates cancer as a dynamic system arising from the integration of multiple regulatory layers, including genomic instability, signaling networks, post-transcriptional regulation, metabolic programs, and the tumor microenvironment. External selective pressures—such as therapy, immune surveillance, metabolic stress, and temporal evolution—continuously perturb these layers, driving reversible transitions between phenotypic states, including proliferation, invasion/metastasis, dormancy, stemness, and resistance. Within this adaptive landscape, continuous network rewiring drives system behavior, while cell–cell communication coordinates system-wide interactions across states. As tumors adapt, they develop transient, context-dependent vulnerabilities that can be therapeutically exploited by targeting network-level processes.

The contributions collected in this Special Issue collectively illustrate how therapeutic opportunities arise from the intersection of multiple regulatory layers, including DNA repair, intracellular signaling, post-transcriptional regulation, metabolic adaptation, and even methodological context. Taken together, these studies suggest that tumor behavior emerges from the coordinated activity of interconnected processes.

Within this context, synthetic lethality provides a particularly clear example of how vulnerabilities can be therapeutically exploited. By simultaneously targeting compensatory DNA repair pathways, it becomes possible to selectively impair tumor cell survival by exploiting dependencies generated by genomic instability (Barszczewska-Pietraszek et al. Table 1). In this study, inhibition of DNA polymerase theta (Polθ), particularly in combination with PARP1 or RAD52 inhibition and the DNA-damaging agent temozolomide, markedly reduced glioblastoma cell viability, with the most pronounced effects observed when multiple DNA repair pathways were simultaneously inhibited in the presence of induced DNA damage. By contrast, normal astrocytes were consistently less affected, maintaining higher levels of viability across most treatment settings. Importantly, temozolomide alone showed a measurable impact on normal astrocytes, in line with its non-specific mechanism of action as an alkylating agent. However, its combination with Polθ inhibition did not proportionally increase this effect, pointing to a differential response between tumor and normal cells rather than complete selectivity. At the same time, the residual impact on normal cells remains relevant and, as noted by the authors, requires careful consideration in the context of therapeutic translation. More broadly, these findings highlight a key concept: therapeutic vulnerabilities are not fixed properties of tumor cells but arise as a consequence of perturbation. When DNA repair networks are disrupted and cells are forced to adapt under stress, new functional dependencies emerge that can be therapeutically exploited. In this sense, treatment itself contributes to reshaping the landscape of vulnerability, revealing targets that would not be apparent under baseline conditions.

At the same time, major signaling pathways underscore the inherent robustness of cancer systems. The MAPK cascade, for example, illustrates how feedback loops and pathway reactivation sustain tumor survival despite targeted inhibition, emphasizing the intrinsic limitations of single-agent approaches and supporting the rationale for combinatorial therapeutic strategies (Levy et al. Table 1). Consistently, experimental evidence shows that vertical targeting of multiple nodes within the MAPK pathway can enhance therapeutic efficacy and partially overcome acquired resistance (Kosnopfel et al. Table 1). In particular, in BRAF-mutant melanoma models, the addition of ERK inhibition to BRAF/MEK-targeted therapy improved growth suppression and promoted apoptotic responses, including in resistant settings, highlighting the value of extending inhibition to downstream components of the pathway. In this context, MEK signaling emerges as a central regulator of proliferation and survival, frequently dysregulated across multiple tumor types, including glioblastoma, where therapeutic options remain limited. Although MEK inhibition has shown encouraging activity in preclinical and early clinical settings, its durability is often constrained by adaptive resistance, ultimately reinforcing the need to move beyond single-pathway inhibition toward coordinated, multi-level targeting strategies.

Beyond genetic and signaling layers, post-transcriptional regulation emerges as a critical determinant of tumor behavior. RNA-binding proteins such as IMP2 (Insulin-like growth factor 2 mRNA-binding protein 2) regulate the stability and translation of oncogenic transcripts, thereby influencing metabolism, immune response, and tumor progression, and representing an additional layer of therapeutic opportunity (Das et al. Table 1). IMP2, in particular, acts as a multi-level regulator integrating metabolic and oncogenic pathways through the control of m6A-modified RNAs. Its upregulation, often driven by genomic amplification or transcriptional overexpression, has been associated with tumor progression and poorer clinical outcomes across multiple cancer types, supporting its potential as both a biomarker and a therapeutic target. Beyond tumor-intrinsic processes, IMP2 also contributes to immune modulation, including macrophage polarization and tumor-immune evasion, linking post-transcriptional regulation to broader aspects of tumor adaptation. Notably, its functional role in metabolic pathways has also been associated with metabolic diseases, further underscoring its relevance as a context-dependent regulator of cellular states. Taken together, these findings highlight how post-transcriptional mechanisms do not simply fine-tune gene expression but actively shape tumor phenotypes, revealing additional layers of vulnerability that complement those arising from genetic and signaling alterations.

Importantly, tumor heterogeneity should be interpreted not only through molecular pathways but also across population-specific contexts. Molecular signatures linked to tumor aggressiveness, including the coordinated activation of focal adhesion kinase and growth factor receptor networks (e.g., PTK2, PTK2B, EGFR, PDGFRB, NGFR, and, in male patients, also CXCR1), emerge in glioblastoma from Puerto Rican Hispanic patients, whereas these patterns are not recapitulated in non-Hispanic cohorts, thereby pointing to ethnicity as a critical and often underappreciated determinant of tumor behavior (Porter et al. Table 1).

In addition, studies in ovarian cancer models using a targeted anticancer compound provide insight into the dynamic response of cancer stem cell (CSC) populations. Treatment of ovarian tumor cell lines induces a robust apoptotic response while selectively reducing CSC abundance relative to non-CSC populations, and is accompanied by coordinated changes in glycosphingolipid composition and metabolic pathways. Notably, membrane lipid remodeling emerges as a key component of this response, suggesting an integrated reprogramming of tumor cell states with implications for tumor persistence and therapeutic resistance (Odak et al. Table 1).

From a systems perspective, resistance should no longer be interpreted as a single event but rather as a stable state emerging from regulatory network dynamics. Computational modeling of the PTENP1/miR-21/PTEN axis, built by integrating evidence across multiple tumor types, including breast cancer, hepatocellular carcinoma, and oral squamous cell carcinoma, shows that cellular responses to DNA damage do not follow a linear trajectory but instead evolve within a landscape of possible states defined by the underlying gene regulatory network. Cells tend to converge toward discrete and stable endpoints, or attractor states, which represent self-sustaining configurations of network activity. Notably, drug resistance and epithelial-to-mesenchymal transition (EMT) emerge as dominant attractors, indicating that these phenotypes correspond to preferred and robust outcomes under stress conditions. These observations are consistent with the well-established “go or grow” dichotomy in glioblastoma, where inhibition of proliferative programs can be accompanied by enhanced migratory and invasive behavior (Levy et al. Table 1), reflecting a shift between distinct phenotypic states. This systems-level view highlights the need to disrupt network dynamics, rather than individual signaling nodes, to effectively overcome resistance (Gupta et al. Table 1).

Finally, an additional layer of complexity arises from methodological variability. Studies evaluating biobanking procedures demonstrate that pre-analytical factors, including plasma isolation techniques, can profoundly influence the molecular composition of samples and systematically bias downstream analyses. Even in the presence of preserved inter-individual variability, such methodological differences can alter absolute measurements and affect data comparability across studies, underscoring the critical need for harmonized, fit-for-purpose protocols and rigorous reporting standards in translational research (Piccotti et al. Table 1).

Taken together, these contributions converge toward a unifying principle in which tumor behavior is increasingly understood as the product of dynamic interactions across multiple regulatory layers. This perspective calls for a conceptual shift: from targeting individual molecules to perturbing adaptive systems. It requires integrative strategies capable of linking experimental biology, computational modeling, and clinical translation, with the aim of anticipating, rather than merely reacting to, tumor evolution.

Ultimately, cancer should not be regarded as intrinsically intractable, but as a dynamic system whose apparent robustness conceals exploitable fragilities. Thus, the key challenge lies not in identifying additional targets but in understanding and disrupting the adaptive logic that sustains malignancy.

## Figures and Tables

**Figure 1:**
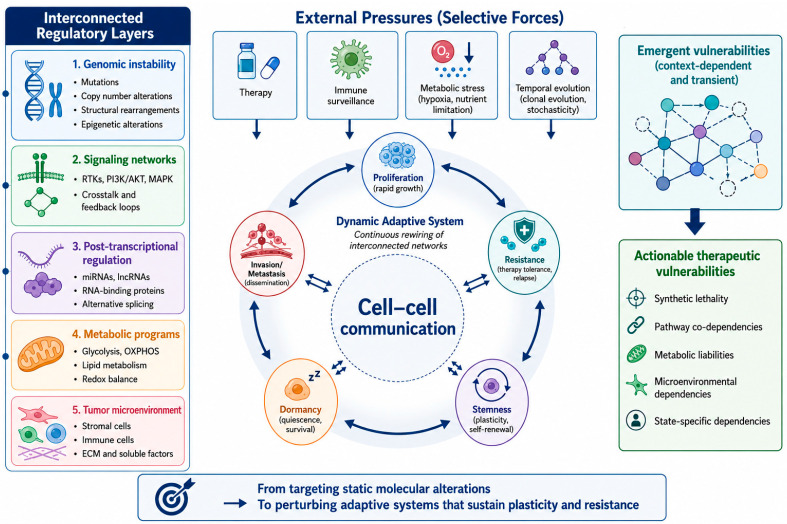
Cancer as a dynamic adaptive system: emergent vulnerabilities across regulatory layers.

**Table 1:** List of the articles present in this Special Issue.

First Author	Reference
Barszczewska-Pietraszek, G.	Barszczewska-Pietraszek et al. *Int. J. Mol. Sci.* 2024, 25, 9134. https://doi.org/10.3390/ijms25179134
Levy, A.S.	Levy et al. *Int. J. Mol. Sci.* 2025, 26, 6875. https://doi.org/10.3390/ijms26146875
Kosnopfel, C.	Kosnopfel et al. *Int. J. Mol. Sci.* 2025, 26, 9794. https://doi.org/10.3390/ijms26199794
Das, J.	Das et al. *Int. J. Mol. Sci.* 2025, 26, 2415. https://doi.org/10.3390/ijms26062415
Porter, T.	Porter et al. *Int. J. Mol. Sci.* 2024, 25, 4947. https://doi.org/10.3390/ijms25094947
Odak, Z.	Odak et al. *Int. J. Mol. Sci.* 2024, 25, 6954. https://doi.org/10.3390/ijms25136954
Gupta, S.	Gupta et al. *Int. J. Mol. Sci.* 2024, 25, 8264. https://doi.org/10.3390/ijms25158264
Piccotti, F.	Piccotti et al. *Int. J. Mol. Sci.* 2025, 26, 10281. https://doi.org/10.3390/ijms262110281

